# Mucus and Mucins: The Underappreciated Host Defence System

**DOI:** 10.3389/fcimb.2022.856962

**Published:** 2022-06-14

**Authors:** Yong Hua Sheng, Sumaira Z. Hasnain

**Affiliations:** ^1^ Immunopathology Group, Mater Research Institute−The University of Queensland, Translational Research Institute, Brisbane, Qld, Australia; ^2^ Australian Infectious Diseases Research Centre, The University of Queensland, Brisbane, Qld, Australia

**Keywords:** mucins, mucus, mucosal epithelial cells, pathogen, microbes, barrier integrity, infection, host defence

## Abstract

The mucosal surfaces that form the boundary between the external environment and the underlying tissue are protected by a mucus barrier. Mucin glycoproteins, both secreted and cell surface mucins, are the major components of the barrier. They can exclude pathogens and toxins while hosting the commensal bacteria. In this review, we highlight the dynamic function of the mucins and mucus during infection, how this mucosal barrier is regulated, and how pathogens have evolved mechanisms to evade this defence system.

## Introduction

The mucus layer, which coats surfaces exposed to the external environment, is a physicochemical barrier that permits the permeation of nutrients and immune factors and excludes toxins and pathogens. Despite being a dynamic, highly organised arm of the innate immune system, the mucus barrier has largely been underappreciated in infectious diseases. Mucins form the critical structural component of the mucosal barrier (Thornton and Sheehan, 2004). O-Glycosylation accounts for up to 80% of the mass of mucins (Thornton et al., 2008). Mucins are divided into two main subfamilies: the cell surface mucins, which anchor to the cell membrane and provide a carbohydrate-rich covering, and the secreted mucins, which give the mucus gel its viscous properties. Evolutionary studies suggest that mucins are ancient, with mucin-like glycoproteins or domains identified in viruses, parasites, and fungi (Freire et al., 2003; Wang et al., 2003; Buscaglia et al., 2006; Lang et al., 2007). In this review, we highlight the critical role of mucins in regulating microbial interactions at the respiratory and intestinal surfaces during homeostasis. In addition, we discuss the critical role of immune-driven changes in mucins in the innate immune response against mucosal pathogens.

## Mucins of the Glycocalyx

**Figure 1:**
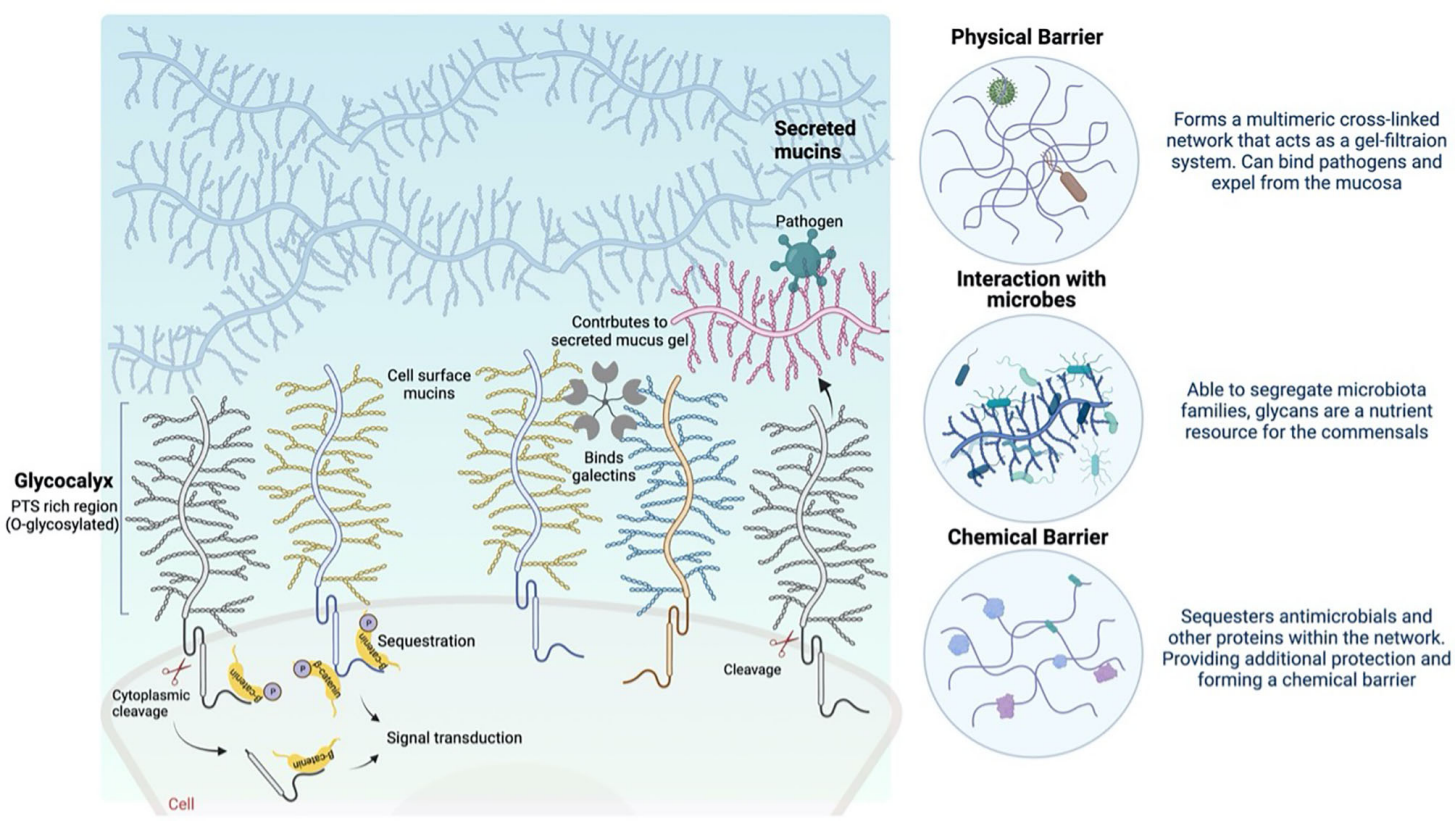
Schematic depicting the role of cell surface and gel-forming mucins at the mucosal barrier (created with BioRender).

The glycocalyx is the carbohydrate-rich layer that covers the mucosal epithelial cells. It contains high amounts of cell-anchored mucin glycoproteins, glycosaminoglycans, and other glycoproteins. Cell surface mucins comprise a large extracellular O-glycosylated domain, which can form long extended and rigid structures at the cell surface. These structures are confronted by pathogens that overcome the secreted mucus layer as they reach the mucosal cell surface. The expression of cell surface mucins and the composition of the glycan structures on these mucins differ depending on the glycosyltransferases at the different tissue sites (**Table 1**) and with infection and inflammation (Jensen et al., 2010). The complex and distinct differences in cell surface mucin expression and oligosaccharide structure can dictate the molecular composition of the epithelial cell surfaces, including pH, ion concentration, enzymatic activity and hydration, and the composition of microbes in the lung and intestine. The extracellular glycosylated domain can dissociate from the cell surface, mediated *via* proteases, after binding to a pathogen as part of a defence mechanism (Parry et al., 2001; Backstrom et al., 2003). Moreover, the cytoplasmic domains of the mucins serve as cell surface receptors and sensors (Sheng et al., 2017; Sheng et al., 2019). Signal transduction, including β-catenin and γ-catenin signalling, through the cytoplasmic domain in response to external stimuli can influence inflammatory responses, proliferation, differentiation, and apoptosis, as discussed below (summarised in **Figure 1**) (Singh and Hollingsworth, 2006). The cell surface mucins surround the cilia in the lung, forming a periciliary layer (PCL), which is essential for the lubrication of the ciliary beat and the movement of mucus through the airways (Ridley and Thornton, 2018). Cell surface mucins are associated with key signal transduction pathways and associated cell surface physical protection, which is an essential part of homeostasis. Cell surface mucins are disrupted in metastatic disease, infection, and inflammation, which correlates with enhanced pathology (Sheng et al., 2017; Sheng et al., 2019).

**Table 1:** Expression of mucins throughout the body.

Tissue	Cell surface mucins	Secreted gel-forming mucins	References
*Respiratory tract*			
Trachea	MUC1 MUC4 MUC16	MUC5AC MUC5B MUC19	Hattrup and Gendler (2008), Thornton et al. (2008)
Bronchus	MUC1 MUC4 MUC16	MUC5AC MUC6	
Alveoli	MUC1 MUC4 MUC16	MUC2	
*Gastrointestinal tract*			
Oral cavity	MUC1 MUC4 MUC16	MUC5B MUC7* MUC19	McGuckin et al. (2011)
Stomach	MUC1 MUC16	MUC5AC MUC6	
Small Intestine	MUC1, MUC3A MUC3B MUC4 MUC12 MUC13 MUC15 MUC16 MUC17	MUC2	
Colon	MUC1, MUC3A MUC3B MUC4 MUC12 MUC13 MUC15 MUC16 MUC17	MUC2 MUC5AC MUC6	

*Non-oligomerizing mucin.

### Function of Cell Surface Mucins

#### Physical Barrier

Recent studies have suggested that cell surface mucins contribute to the maintenance of the mucosal barrier integrity by preventing adhesion of foreign debris, cells, or pathogens onto the mucosal surface epithelia (Argueso et al., 2006; Imbert et al., 2006; Blalock et al., 2007; Corrales et al., 2011). The large protein core, dense O-glycosylation, charge repulsion, and hydration results in steric hindrance, making the cell surface mucins relatively rigid structures (Gipson and Inatomi, 1998; Cone, 2009; McGuckin et al., 2011; Petrou and Crouzier, 2018), which remain unaltered despite movement and shearing at the mucosal surfaces. Protruding considerably further from the cell surface (e.g., MUC1 can extend >200 nm), the cell surface mucins are thought to prevent adhesion of the secreted mucus layer directly to the epithelial cell surface. Sumiyoshi et al. demonstrated that the anti-adhesive character of mucin O-glycans at the apical surface of corneal epithelial cells was caused by the repulsive negative charge interactions between secreted and cell surface mucins (Sumiyoshi et al., 2008). In addition, the mucins anchored to the cell surface have the ability to bind to glycan ligands, such as galectin-3, which can generate molecular matrices and facilitate mucin assembly, reinforcing the physical barrier on the epithelial cell surface (Argueso et al., 2009; Gipson et al., 2014).

#### Regulation of Signal Transduction

Cell surface mucins regulate the signal transduction through both extracellular and cytoplasmic domains. It has been suggested that cell surface mucins sense the environment through their extracellular domain and signal through their intracellular domain (Singh and Hollingsworth, 2006). Several cell surface mucins have epidermal growth factor (EGF)-like motifs in their extracellular domain adjacent to the cell membrane. These EGF-like motifs interact with a wide range of receptors, prompting cell proliferation following epithelial injury in the normal tissue, and in cancer progression to metastasis (Singh and Hollingsworth, 2006). MUC4 has at least two EGF-like domains, which can activate ErbB2 and ErbB3 receptors responsible for epithelial cell proliferation and apoptosis (Singh and Hollingsworth, 2006). MUC1 and MUC13 can regulate chemokine secretion from intestinal epithelial cells. The cytoplasmic domain of MUC1 contains multiple phosphorylated tyrosine residues that can activate intracellular signalling pathways, such as the ERK1/2 pathway in airway epithelial cells (Wang et al., 2003). It can also interact with β-catenin, catenin p120, ER-α, p53, and nuclear factor kappa B (NF-ĸB) to convey specialised signalling in response to conditions at the cell surface, such as binding with pathogens and changes in pH (Singh and Hollingsworth, 2006). MUC13 can enhance NF-ĸB activation and prevent cell death, which can advance tumour formation and progression (Sheng et al., 2017).

## Secreted Mucus Barrier

Gel-forming mucins, namely, MUC2, MUC5AC, MUC5B, MUC6, and MUC19, similar to cell surface mucins, have a large protein core, with a repetitive amino acid sequence (PTS) that is proline-, threonine-, and serine-rich region and heavily O-glycosylated. O-Glycosylation is the key element responsible for the hydrophilic character of the secreted mucins and allows expansion and extension of the core. The individual mucin subunits can then form dimers *via* disulphide bonds and the C-terminus cysteine knot domains. Dimers can assemble into multimers *via* the intermolecular disulphide bonds at the N-terminus von Willebrand domains. The oligomeric nature of each mucin is thought to be different; MUC2 is believed to oligomerise in a trimeric form (Godl et al., 2002), while MUC5B oligomerises in a linear form (Hughes et al., 2019). Glycosylation, along with non-mucin proteins, calcium content, and covalent (disulphide bonds) and non-covalent hydrogen bonds, are all determinants of the viscoelastic and chemical properties of the mucus gel (Ridley et al., 2014; Meldrum et al., 2018).

### Function of Gel-Forming Mucins

#### Lubrication and Physical Barrier

Once secreted by goblet cells, submucosal gland cells, or serous cells, gel-forming mucins form a highly hydrated mucus gel and contribute to the lubrication of the epithelial cell surfaces. The hydrophilic nature of the mucins is thought to reduce shear stress at the epithelial surface: for instance, peristalsis and movement of stool through the intestine (McGuckin et al., 2011; Petrou and Crouzier, 2018). This polymeric network of secreted mucins acts similarly to a gel filtration system. *Ex vivo* measurements using fluorescent probes have shown that large molecules such as pathogens cannot pass through, while smaller molecules like antimicrobial peptides can easily penetrate the mucus gel (Hasnain et al., 2010; Gustafsson et al., 2012).

In the intestine, the mucus layer, mainly composed of MUC2, forms an inner adherent layer and an outer loose layer. The inner adherent layer is “sterile,” composed of MUC2 multimers that are presumably tightly packed to provide protection from the commensal flora (Hansson and Johansson, 2010; Gustafsson et al., 2012). Hansson et al. hypothesised that MUC2 multimers are organised as sheets that interact with the epithelial cell layer and cell surface mucins; however, X-ray crystal structure of MUC2 multimerisation module suggests that non-covalent and covalent interactions form a lateral network (Javitt et al., 2019). The outer mucus layer is exposed to proteases and bacteria, which enables it to become less dense. This is also important for maintaining homeostasis, as the faecal material in the intestine generates mechanical stress (McGuckin et al., 2011). The respiratory mucus layer is more complex. It comprises two gel-forming mucins, MUC5AC and MUC5B, which only form homo-multimers in the lung. Although MUC5B expression was mainly thought to be restricted to submucosal glands, recent data show that the distal airway superficial epithelium is the predominant site for MUC5B expression, while MUC5AC expression is concentrated in the proximal airways (Bonser and Erle, 2017; Hancock et al., 2018; Hughes et al., 2019; Okuda et al., 2019). Once secreted into the airway lumen, these mucins can non-covalently cross-link to form a physical barrier that can be easily moved by the cilia (McGuckin et al., 2011; Bonser and Erle, 2017).

#### Clearing Molecules

The secreted gel-forming mucins can trap allergens and debris to facilitate their clearance from the mucosal surface (Rubin, 2002). This role is assisted by the incredible diversity of the carbohydrate side chains, which enhances the possibility of pathogens binding to the mucus (Thornton and Sheehan, 2004). While it has been reported that hydrophilic contaminants are easily repulsed by the mucus gel, weakly polar contaminants are trapped in the mucus gel (Sharma, 1993). Contaminants, including pathogens and allergens, are then eliminated along with the mucus (Gipson and Argueso, 2003; Dartt and Masli, 2014). In a patient with congenital loss of MUC5B, Costain et al. (2022) highlight the key role of mucins and demonstrate impaired mucociliary clearance and increased inflammatory macrophage infiltrate in sputum (Costain et al. 2022).

### Antimicrobial Agents

The secreted mucus network provides a scaffold for antimicrobial molecules and antibodies. The retention of these molecules within the physical barrier functions as a chemical barrier against commensals and pathogens. Mucins have been shown to have direct potent anti-pathogenic activity. For example, Muc5ac upregulated in the intestine during nematode infection directly reduced ATP levels in the nematodes (Hasnain et al., 2011). Muc2 acts as a chemoattractant; it binds to *Campylobacter jejuni* and limits its growth (Tu et al., 2008).

Together, the complex mucin structure, the oligomerisation into a network, and the chemical sequestration of antimicrobials in the intestine and lung provide an efficient physical barrier against pathogens. However, many pathogens have developed ways to overcome the key protective properties of the mucin and mucus barrier.

## Mucins–Microbiota Interaction

There is a tremendous number of microorganisms, termed microbiota, which reside at different mucosal surfaces. The intestinal microbiota are well studied (Corfield, 2018; Schroeder, 2019). However, recently, commensal bacteria in the lung have gained attention, albeit the number of microorganisms is lower in the lung at baseline compared with the intestine (Enaud et al., 2020). Microbiota have a mutualistic relationship with their host, and the interaction of microbiota and mucins appears to be bidirectional at least in the intestine and reproductive tract (Schroeder, 2019). Microbiota is generally considered beneficial; however, the vast number of microorganisms also form a permanent threat to the host. Thus, to prevent direct interaction of microbes with the epithelial cell layer and their translocation across the mucosal barrier, the host has developed effective physical and chemical defence mechanisms, including the secreted mucus barrier and the glycocalyx that covers the epithelium (Corfield, 2018). It is difficult to dissect in diseases such as inflammatory bowel disease or metabolic syndrome whether it is the imbalance of microbiota or alterations in mucins that drive the pathology. Although deficiency in microbiota (germ-free animals) and alterations or absence of mucins in rodent models have been shown to individually enhance susceptibility to disease, both are accompanied by changes to the other (Hill et al., 1990; Enss et al., 1996; Wang et al., 2021).

The significant individual variation in the distribution of microbiota is thought to be determined by polymorphic host glycosylation (Turnbaugh et al., 2009). The host-specific glycan repertoire of mucins is important for the regulation of the composition, growth, and behaviour of the microbiota. In return, maturation, function, and glycosylation of mucins are influenced by the gut microbiota (Schroeder, 2019). For instance, short-chain fatty acids, produced because of bacterial fermentation of fibres, can regulate the production of mucins. Additionally, commensal mucolytic bacteria maintain the appropriate turnover of the outer mucus layer, which favours the host by competitively excluding pathogens. For some bacteria, mucins can be virtually their only energy source (Johansson and Hansson, 2016), and therefore, the O-glycans can influence the repertoire of microbiota present. While mucin glycosylation can dictate the composition of the microbiota, the microbiota can influence epithelial cell function, metabolism, and proliferation (Ashida et al., 2011). However, there are still gaps in our understanding of the host dictating the microbial diversity and population. Microbiota are crucial for the development of an effective immune system, as supported by the deficiency in mucus and several immune cell types displayed by germ-free animals. There are fewer intestinal goblet cells, and there is decreased storage of mucin granules in germ-free conditions compared to normal conditions (Hill et al., 1990; Enss et al., 1996). In addition, there is decreased expression of some antimicrobial molecules, including angiogenin 4 and REGIIIγ (Hooper et al., 2003; Cash et al., 2006). A lack of expansion of the CD4^+^ T-cell population is also reported in germ-free animals, which can be reversed by the treatment with polysaccharide A from *Bacteroides fragilis* (Mazmanian et al., 2005).

## Understanding the Interaction Between Pathogen and Mucins

**Figure 2:**
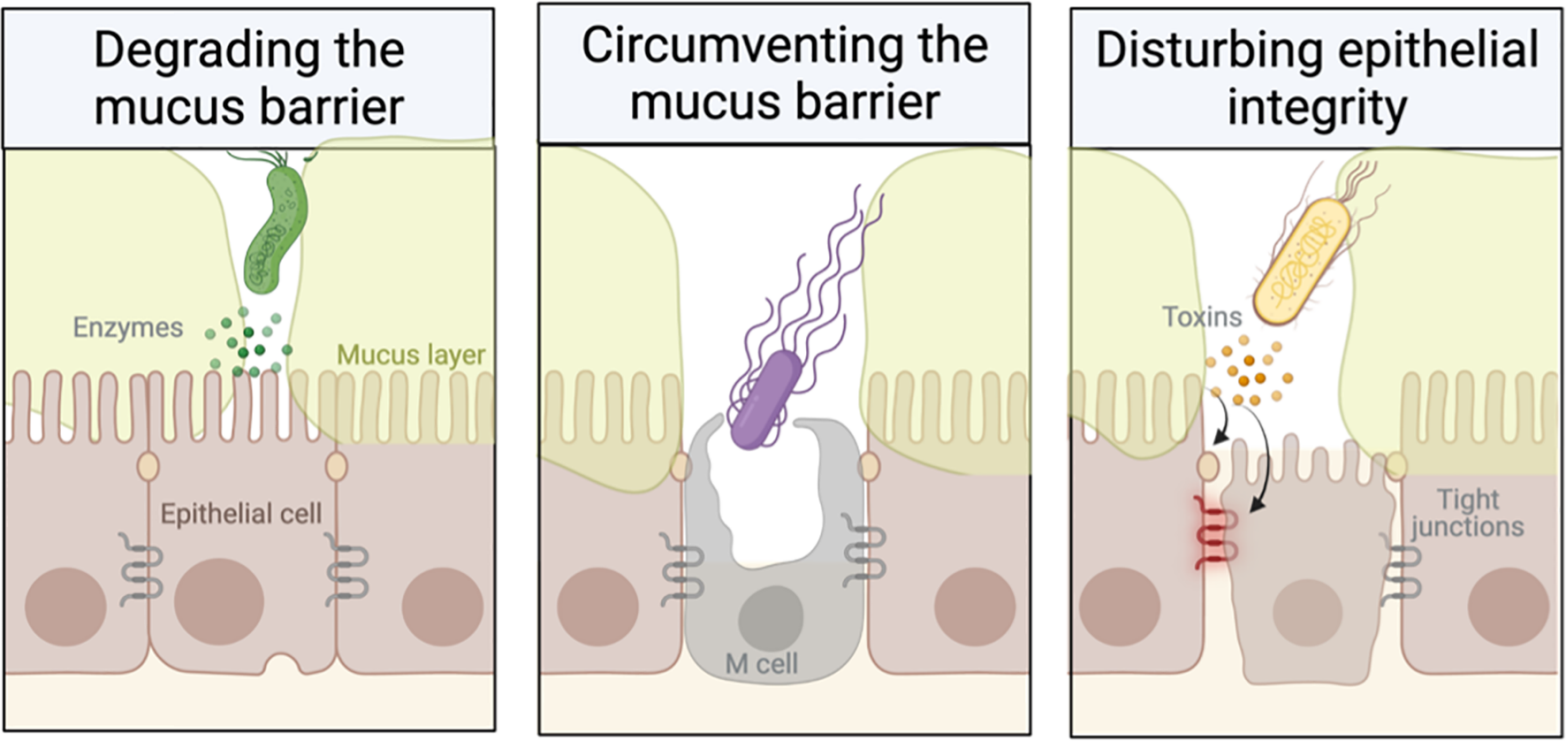
Strategies employed by pathogens to overcome the mucus barrier (created with BioRender).

Mucin glycoproteins are a critical element of the mucosal barrier to infection. This barrier is dynamic and responsive to elements of both innate and adaptive immunity. It uses multiple defence mechanisms against microbes, including secreted mucus, the apical glycocalyx, and epithelial tight junctions. However, mucosal pathogens can efficiently infect the mucosa using a wide range of specific strategies that allow them to subvert or avoid the mucin barrier in the gut. Indeed, bacterial pathogens have evolved into highly sophisticated protein export systems, which have been discussed previously (Ashida et al., 2011) and will not be examined here. Instead, we will focus on the properties of mucins that confer protection and on mechanisms used by pathogens to evade this barrier (**Figure 2**).

Most of our understanding comes from animal models, since the mucosal environment is extremely complex and impossible to fully recreate *in vitro*. However, in the past decade, primary organoid culture systems have provided the field with a model system that can replicate part of the complexity of the cellular and secreted mucus barrier (comprehensively reviewed by Han et al., 2021). Here, we mainly focus on the studies from animal models, which capture the cellular and secreted barrier along with an intact immune system to highlight how mucins are functionally important in limiting infection and inflammation.

### Physical Barrier Function

Goblet cell hyperplasia and increased mucin expression or secretion have been reported in several mucosal infections, including nematode, *Citrobacter*, and *Pseudomonas* infection (Tu et al., 2008; Bergstrom et al., 2010; Hasnain et al., 2010; Umehara et al., 2012). This supports the “mucus-trap” hypothesis, which suggests that the host uses increased mucin release to physically trap pathogens within the mucin network (Miller, 1987; Bergstrom et al., 2010). Subsequently, the pathogens are eliminated with the movement of the mucus layer, e.g., through peristalsis or coughing (Miller, 1987; Bergstrom et al., 2010). Reduced mucus permeability in *Muc2^−/−^* mice demonstrates the importance of mucins in determining the physical properties of the barrier (Hasnain et al., 2010). Many bacteria have the ability to produce a series of hydrolytic enzymes, which degrade the mucus glycans. These enzymes, such as glycosulphatases, sialidases, and sialate O-acetylesterases, degrade the mucin oligosaccharides, exposing the mucin peptide backbone to proteases while also removing decoy carbohydrates for microorganism adhesins (Kato and Ishiwa, 2015). Proteolytic cleavage of mucins causes disassembly of the oligomerised mucin macromolecules, resulting in greatly reduced mucus viscosity and diffusion of the mucus. The protozoan parasite *Entamoeba histolytica* can proteolytically cleave MUC2 to disrupt the colonic mucus by breaking down the macromolecular structure and invading the underlay epithelium (Lidell et al., 2006). Pathogens can secrete proteases to degrade Muc2, such as *Trichuris muris* nematodes (Henderson et al., 1999; Hasnain et al., 2012). Other pathogens secrete zinc metalloproteases that non-specifically cleave mucin-O-glycosylated proteins (Silva et al., 2003; Grys et al., 2005). Mucus degradation is not limited to pathogens. Some commensal bacteria, like *Akkermansia muciniphila* and *Bacteroides bifidum*, are also mucolytic and can use mucin glycoproteins as an energy source (Macfarlane and Gibson, 1991; Png et al., 2010). These microorganisms are typically strictly anaerobic and do not penetrate the inner mucus layer (McGuckin et al., 2011). In conjunction with mucus degradation, motility is also important for enteric pathogens to break through the mucus barrier. Many enteric pathogens have evolved strategies to infect the host by avoiding the mucus barrier. Pathogenicity with disrupted flagellar function is reduced, highlighting the importance of motility in disease (Ramos et al., 2004). For example, disrupting flagella in *Helicobacter pylori* greatly reduced its ability to promote infection (Ottemann and Lowenthal, 2002).

### Protecting Epithelial Integrity

Enteric pathogens commonly use the type III secretion system (T3SSs) to deliver the effector proteins (also referred to as toxins) to the cytoplasm of the host cell (Ashida et al., 2011) and, consequently, alter the expression/production of mucins. These toxins can directly cause cell death, growth inhibition, cell cycle arrest, modulation of inflammatory signalling, and disruption of tight junctions. This dysregulation can result in shifts in the microbial population, which can compromise the mucosal epithelium integrity (Dalby et al., 2006; Wroblewski et al., 2009). Tight junction disruption during pathogen infection often causes barrier failure, which subsequently allows the translocation of commensal bacteria across the damaged epithelial lining, resulting in inflammation (Walk et al., 2010; Ashida et al., 2011). Pathogens can disrupt several signalling pathways and expose the vulnerable lateral cell membranes that are not protected by the mucins, enabling the pathogens to penetrate deeper into the mucosal tissues (McGuckin et al., 2011). Examples of bacteria that interfere with tight junctions include enteropathogenic *Escherichia coli* (Goosney et al., 2000), *Shigella flexneri* (Sakaguchi et al., 2002), *Salmonella* (Boyle et al., 2006), *Vibrio parahaemolyticus* (Yarbrough et al., 2009), and *H. pylori* (Wroblewski et al., 2009).

### Anti-Inflammatory Effects

#### Cell Surface Mucins

MUC1 has been suggested to play an anti-inflammatory role during *Pseudomonas aeruginosa* respiratory infection, as *Muc1^−/−^* mice are more susceptible to infection in a repetitive *Pseudomonas* infection model (**Table 2**) (Lu et al., 2006). Colonisation is associated with stronger immune responses (Lu et al., 2006), including increases in tumor necrosis factor alpha (TNFα) and interleukin (IL)-8 in bronchoalveolar lavage fluids compared with wild-type (WT) mice (Umehara et al., 2012). Deficiency in Muc1 also predisposed mice to infection with the gastrointestinal pathogen *C. jejuni* and the gastric pathogen *H. pylori* (**Table 2**) (McAuley et al., 2007; McGuckin et al., 2007). In cases of acute infection with *C. jejuni*, *Muc1^−/−^* mice rapidly develop systemic infection, suggesting that Muc1 limits this pathogen penetration through the mucosal barrier, and Muc1 also modulates the epithelial cell response to a bacterial genotoxin (McAuley et al., 2007). Similarly, *Muc1^−/−^* mice showed a five times higher density of infection (increased colony forming units) as early as 1 day after oral gavage with *H. pylori* compared with WT mice (Sheng et al., 2020). Furthermore, more severe chronic inflammation was observed in *Muc1^−/−^* mice after exposure to *H. pylori*, demonstrating that this cell surface mucin can modulate the inflammatory response to chronic infection (McGuckin et al., 2007). *Muc1^−/−^* mice also develop severe pathology in response to influenza A virus infection (**Table 2**) (McAuley et al., 2017). In the absence of *Muc1*, the kinetics of the infection are altered. Animals reach maximal influenza A viral load earlier than WT mice and also display enhanced inflammatory response to the infection (McAuley et al., 2017). Similarly, a higher viral titre was detected in *Muc1^−/−^* mice compared to WT mice in response to intranasal inoculation of murine adenovirus type 1 (MAV-1) (McAuley et al., 2017), suggesting that the Muc1 may protect against MAV-1 respiratory infections (Nguyen et al., 2011). MUC1 has been shown to have an anti-inflammatory response during respiratory syncytial viral infection *in vitro* (Li et al., 2010).

**Table 2:** Mucin mouse models for study infection.

Tissue	Animal model	Pathogens	Cell type	Susceptibility to infection	Inflammatory response	References
Stomach	*Muc1^−/−^*	*Helicobacter pylori*	Gastric epithelial cells, macrophage	Increased	Increased	McGuckin et al. (2011)
	*Muc5ac^−/−^*	*Helicobacter pylori*	Gastric epithelial cells	Increased	Increased	Muthupalani et al. (2019)
Intestine	*Muc1^−/−^*	*Campylobacter jejuni*	Intestinal epithelial cells	Increased	Increased	McAuley et al. (2007)
	*Muc2^−/−^*	*Escherichia coli*	Intestinal epithelial cells	Increased	Decreased	Bergstrom et al. (2010)
	*Muc2^−/−^*	*S.Tm*	Epithelial cells	Increased	Increased	Zarepour et al. (2013)
	*Muc2^−/−^*	*T. muris*	Intestinal epithelial cells	Increased	Increased	Hasnain et al. (2010)
Lung	*Muc1^−/−^*	*Pseudomonas aeruginosa (Pa)*	Tracheal epithelial cells, alveolar macrophages	Decreased colonisation	Increased	Lu et al. (2006)
	*Muc1^−/−^*	*Pa* (4xrepeated)	Lung epithelial cells	Increased	Increased	Umehara et al. (2012)
	*Muc1^−/−^*	*Influenza A virus*	Airway epithelial cells	Increased	Increased	McAuley et al. (2017)
	*Muc1^−/−^*	*Murine adenovirus type I*	Airway epithelial cells	Increased	ND	Nguyen et al. (2011)
	*Muc5ac^−/−^*	*Respiratory syncytial virus*	Airway epithelial cells	Increased	Increased	Cho et al. (2021)

ND, No difference.

Muc3 (the murine orthologue of human MUC17) and Muc13 are two of the most abundant cell surface mucins in the normal intestinal tract. Interestingly, Muc3 may play a role in wound healing in acute chemical-induced [dextran sodium sulphate (DSS)] colitis: intrarectal administration of recombinant cysteine-rich domains of Muc3 accelerated cell migration and reduced apoptosis in the distal colon (Ho et al., 2006). Corroborating this anti-inflammatory effect, we have demonstrated that Muc13-deficient mice have increased susceptibility to DSS-induced colitis, increased local inflammatory cytokine production, and increased epithelial cell apoptosis (Sheng et al., 2011). Overall, there is strong evidence supporting a critical role of cell surface mucins in protection against inflammation by modulating growth and inhibiting apoptosis of epithelial cells during wounding and repair.

#### Gel-Forming Mucins

Transgenic animals lacking secreted gel-forming mucins have also demonstrated their anti-inflammatory effects. *Muc5ac^−/−^* mice have higher *H. pylori* colonisation densities compared with WT animals at 16 weeks post-infection, along with a significant reduction in gastric *Tnfα* and *Il-17a* (**Table 2**) (Muthupalani et al., 2019). Furthermore, *H. pylori*-infected *Muc5ac^−/−^* mice had significantly lowered gastric corpus mucous metaplasia at 16 weeks post-infection (wpi) and 32 wpi compared with WT mice. Our work has shown that *de novo* intestinal goblet cell expression of Muc5ac in the intestine is critical in the protection against *Trichuris* nematode. Muc5ac but not Muc2 reduced nematode ATP levels and was responsible for the expulsion of the nematode. These studies demonstrate a protective role for Muc5ac in inhibiting pathogen-associated inflammatory pathology. Significantly greater inflammation and fibrosis by bleomycin were developed in *Muc5ac^−/−^* lungs compared to WT animals (Cho et al., 2021). Airway respiratory syncytial viral (RSV) replication was higher in *Muc5ac^−/−^* than in *Muc5ac^+/+^* during early infection. RSV-caused pulmonary epithelial death, bronchial smooth muscle thickening, and syncytia formation were more severe in *Muc5ac^−/−^* compared to WT mice (Cho et al., 2021).

Muc5b, but not Muc5ac, was shown to be critical in trapping and clearing microbial pathogens through mucociliary clearance in the lung (Roy et al., 2014; Hancock et al., 2018). Muc5b*^−/−^* mice have a significantly dysfunctional inflammatory response, with an increase in neutrophils and eosinophils but an absence of lymphocytes. There was an increased bacterial accumulation, including *Staphylococcus aureus*, in the lung of Muc5b*^−/−^*, which, combined with the lack of lymphocytes, leads to increased mortality (Roy et al., 2014).

MUC2/Muc2 is the main secreted intestinal mucin expressed and secreted by all enteric goblet cells. While acute infection leads to goblet cell hyperplasia and increased Muc2, chronic inflammation in the intestine is associated with goblet cell depletion. *Muc2^−/−^* mice are more susceptible to *Salmonella typhimurium* infection, with increased mortality rates, higher pathogen burdens, and developing significantly higher barrier disruption compared with WT animals (**Table 2**) (Zarepour et al., 2013). Similarly, *Muc2^−/−^* mice show rapid loss of weight and up to 90% higher mortality in response to *Citrobacter rodentium*, a murine attaching–effacing (A/E) pathogen related to diarrheagenic A/E*. coli* (**Table 2**) (Bergstrom et al., 2010). We have shown that expulsion of the *Trichuris* worms from the intestine was significantly delayed in Muc2-deficient mice compared with WT mice (**Table 2**) (Hasnain et al., 2010).

## Conclusion

In this review, we highlight and provide evidence for the mucins at the mucosal surfaces as a key part of our innate immunity. Mucins and in particular the O-glycosylation is thought to dictate the composition of the microbiota, provide essential physical and chemical scaffolds within the mucosal barrier, and are closely interlinked with the adaptive immune system. However, the intricate details of how this is regulated is still unknown. Despite the plethora of literature highlighting the direct and indirect role of mucins in protecting against infectious disease, there is a lack of appreciation and focus on the mucus barrier as a highly responsive arm of the immune system. When considering innate immunity, microbial composition, and adaptive immunity, there should be a push in the mucosal immunology field to develop a multidimensional approach that recognises the mucus barrier (and mucins) as an integral part of the immune response. This will help us address the fundamental gaps in our knowledge, including understanding the mucin structure; its function in health, disease, and during infection; the crosstalk between mucin and microbes; identifying factors that drive changes in mucin O-glycosylation; and the rheological properties of the mucus gel.

## Author Contributions

YS and SH wrote the review and edited the review. SH conceived the idea of the review.

## Funding

YHS is supported by the Mater Foundation and a National Health and Medical Research Council Project Grant (APP116414), GESA Mostyn Family Grant, and GESA Project Grant.

## Conflict of Interest

The authors declare that the research was conducted in the absence of any commercial or financial relationships that could be construed as a potential conflict of interest.

## Publisher’s Note

All claims expressed in this article are solely those of the authors and do not necessarily represent those of their affiliated organizations, or those of the publisher, the editors and the reviewers. Any product that may be evaluated in this article, or claim that may be made by its manufacturer, is not guaranteed or endorsed by the publisher.
